# Multi-scale polarisation phenomena

**DOI:** 10.1038/lsa.2016.11

**Published:** 2016-01-15

**Authors:** Vladimir Kalashnikov, Sergey V Sergeyev, Gunnar Jacobsen, Sergei Popov, Sergei K Turitsyn

**Affiliations:** 1 AIPT, Aston University, Birmingham, West Midlands B4 7ET, UK; 2 Acreo, Electrum 236, SE-16440, Kista, Sweden; 3 Royal Institute of Technology (KTH), SE-1640, Stockholm, Sweden

**Keywords:** fibre Raman amplifier, multi-scale methods, polarisation, stochastic calculations

## Abstract

Multi-scale methods that separate different time or spatial scales are among the most powerful techniques in physics, especially in applications that study nonlinear systems with noise. When the time scales (noise and perturbation) are of the same order, the scales separation becomes impossible. Thus, the multi-scale approach has to be modified to characterise a variety of noise-induced phenomena. Here, based on stochastic modelling and analytical study, we demonstrate in terms of the fluctuation-induced phenomena and Hurst R/S analysis metrics that the matching scales of random birefringence and pump–signal states of polarisation interaction in a fibre Raman amplifier results in a new random birefringence-mediated phenomenon, which is similar to stochastic anti-resonance. The observed phenomenon, apart from the fundamental interest, provides a base for advancing multi-scale methods with application to different coupled nonlinear systems ranging from lasers (multimode, mode-locked, random, etc.) to nanostructures (light-mediated conformation of molecules and chemical reactions, Brownian motors, etc.).

## Introduction

Many processes in nature have different temporal and spatial scales that lead to multi-scale complexity. To describe this complexity on different levels, multi-scale methods have been developed and explored for more than 100 years^1,2,3^. For example, in nonlinear fibre optics, three groups of scales have 13 orders of magnitude separation between the smallest length scale of 1.55 µm and the Southern Cross Cable Network, whose length is approximately 32 500 km. The shortest micron scale is related to the wavelength of light and the core diameter. Thus, Maxwell’s equations have to be explored for characterisation of fibre in the dispersion relations context. The next metre scale corresponds to the fibre beat and correlation lengths, e.g., lengths at which a state of polarisation (SOP) reproduces itself and preserves deterministic evolution. The longest kilometre length scale is the fibre attenuation and gain scale, chromatic dispersion and the Kerr nonlinearity. At this length scale, the Manakov equation is obtained by averaging the randomly varying birefringence^4,5,6^.

To describe the pump and signal SOPs evolution in a fibre Raman amplifier (FRA), different multi-scale averaging techniques have been used^7,8,9,10,11,12,13,14,15,16,17,18,19,20^. Some of them account for the scale of birefringence fluctuations (SBF)^7,8,9,10,11^, whereas others account for the SBF and the scale where the pump and signal SOPs interact^12,13,14,15,16,17,18,19,20^. All of the averaging techniques, stochastic modelling, and experimental study demonstrated polarisation pulling (polarisation trapping) of the signal SOP to the pump SOP^7,8,9,10,11,16,17,18,19,21,22,23,24,25,26,27^.

Along with polarisation pulling, our recent theoretical study^13,14,16,17,19^ revealed an additional phenomenon similar to the fluctuation-induced escape (FIE)^28,29,30,31^, which occurred with an increased polarisation mode dispersion (PMD) parameter *D_p_*^4,5,6,32^. The simplest manifestation of this effect, in the form of the resonance-like increase of the gain fluctuations as a function of the PMD parameter, has been first found theoretically by Lin and Agrawal^12^ and experimentally by Popov and co-workers^24^. Additionally, it has been studied theoretically in detail by Sergeyev and co-workers^13,14,16,17,19^.

Modern fibre Raman-based unrepeatered transmission systems use bidirectional pumping schemes^33^. The co-propagating pump and signal provide a major contribution to the pump-to-signal relative intensity noise (RIN) transfer, which also depends on the PMD value^34^. To study the statistical properties of forward pumped FRA is the key to unlocking the RIN characterisation, which is based on the vector models of FRA, and, thus, to developing efficient vector RIN suppression techniques.

To justify application of previously explored multi-scale techniques for studying statistical properties of co-propagating pump and signal SOPs, we, for the first time, use computer simulation of stochastic differential equations with application of the Klöden-Platen-Schurz algorithm, which provides the fastest convergence (see Supplementary Information). We reveal both the polarisation pulling and resonance-like escape from polarisation pulling in terms of fluctuation-induced phenomena metrics, such as Kramers and intrawell relaxation lengths, gain, root mean square (RMS) gain fluctuations, as well as the spectrum, correlation function, the Hurst parameter and probability distribution function for projecting the signal SOP to the pump SOP. The FRA pump–signal SOPs interaction is defined by the rate of relative rotation of the signal SOP with respect to the pump SOP. According to the results of our analytical study, the stochastic modelling demonstrates that for some PMD parameters, which are typical for the currently used single mode fibres, this rate is on the same scale as the birefringence correlation length. Thus, the rate has to be included in the fast scale group with further averaging to obtain a correct description in the region of resonance-like escape from the polarisation pulling.

## Materials and Methods

### Signal and pump states of polarisation evolution in terms of biased Brownian motion

**Figure 1:**
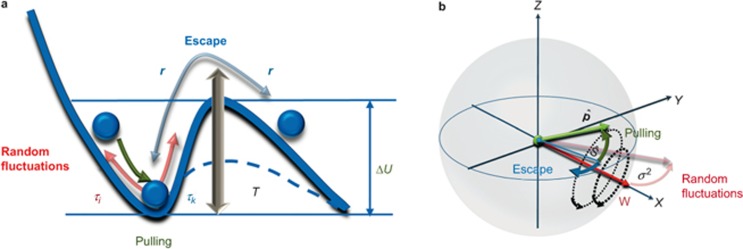
(**a**) Fluctuation-induced phenomena, where the escape probability is controlled by an external periodic force. *ΔU* – potential well, *T* – period of barrier modulation, *r* = 1/τ*_k_* – escape rate, *τ_i_* – intrawell relaxation time, *τ_k_* – residence time (the Kramers time); (**b**) Evolution of the pump 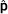 and signal  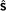 states of polarisation (SOPs) and the local birefringence vector (BV) ***W_i_*** = (2*b_i_*cosθ, 2*b_i_*sinθ,0)*^T^* on the Poincaré sphere. Vectors 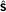 and 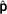 rotate around the local axis ***W*** at rates *b*_p_ and *b*_s_, vector ***W*** rotates randomly in the equatorial plane at the rate *σ* = *L_c_*^−1/2^ (*L_c_* is the correlation length). Anisotropy of fibre Raman amplification, which results in the signal-to-pump SOP polarisation pulling, i.e., builds up a potential well, while relative rotation of the signal SOP, with the rate *b*_p_ – *b*_s_, plays a barrier modulation role, and the random fluctuation of BV defines the noise.

To provide an insight into the FIE phenomena for the fibre Raman amplification, we, first, outline similarities between the SOP evolutions in Raman and biased Brownian motion. The FIE from a metastable state of an excitable system with probability controlled by an external force is a fundamental phenomenon that is inherent in many physical processes, such as, diffusion in crystals, protein folding, activated chemical reactions and many others^28,29,30,31^. Figure 1a demonstrates the escape of the Brownian particle from the bottom of the potential well due to fluctuations and barrier height modulation. The potential well Δ*U* is a source of polarisation pulling (i.e., ‘polarisation trapping’^10^). For the adiabatic forcing case, specifically, when the period of barrier modulation *T* is much longer than the intrawell relaxation time *τ_i_*, FIE takes the form of stochastic resonance (SR), such as, synchronisation between the activated escape events from the potential minimum with a periodic forcing, which results in the maximal signal-to-noise ratio at *T* = *τ_k_* (*τ_k_* is the Kramers time, which characterises the average residence time with respect to the FIE^28,29,30,31^).

The evolution along the fibre length for signal and pump SOP is similar to the evolution of the Brownian particle in the potential well (Figure 1a). As follows from Figure 1b, the pum**p** 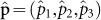 and signal ŝ=(*ŝ*_1_,*ŝ*_2_,*ŝ*_3_) SOPs evolution comprises: (i) signal-to-pump SOP pulling (i.e., polarisation trapping caused by potential well build-up)^7,8,9,10,11,16,17,18,19,21,22,23,24,25,26,27^ caused by the stimulated Raman scattering anisotropy; (ii) barrier modulation caused by the relative rotation of signal SOP with respect to the pump SOP at the rate *b_s_* − *b_p_* (*b_i_* = *π*/*L*_b*i*_, where *L*_b*i*_ is the beat length, *i* = *s*, *p*) around the birefringence vector (BV) ***W_i_*** = (2*b_i_*cosθ, 2*b_i_*sinθ, 0)*^T^*, that randomly fluctuates in the equatorial plane. We assume that the birefringence strength 2*b_i_* is fixed and the orientation angle *θ* is driven by a white-noise process (fixed-modulus model^4,5,6^).


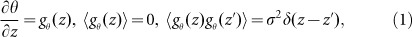


where <…> represents averaging of the birefringence fluctuations along the fibre, δ(z) is the Dirac delta function, and *σ*^2^ = 1/*L_c_* (*L_c_* is the birefringence correlation length). As a result of evolution, the signal wave is amplified and changes its direction as follows:


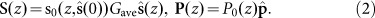


Here, *s*_0_ is part of the signal amplitude that is related to the pump and signal SOPs interaction, 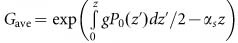 is the averaged Raman gain, *g* is the Raman gain coefficient, *P*_0_ is the pump power at distance *z*, *P*_0_(*z*)=*P_in_* exp (–α*_p_z*); *P_in_* is the input pump power, *α*_s_ and *α_p_* are the signal and pump losses, respectively; *L* is the fibre length.

The part of the Raman gain <*G*>, which is related to the pump–signal SOPs coupling is


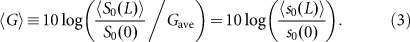


We excluded the averaged Raman gain G_ave_ in Equation (3). This allows us to concentrate on the vector nature of the processes under consideration. If the input pump and signal SOPs are parallel, the Raman gain adopts the maximum value, and, if the SOPs are orthogonal, then the Raman gain adopts the minimum value^7,8,9,10,11,12,13,14,15,16,17,18,19,20,21,22,23,24,25,26,27^. The gain difference is referred to as the polarisation-dependent gain (PDG) and is defined as follows^7,8,9,10,11,12,13,14,15,16,17,18,19,20,21,22,23,24,25,26,27^:


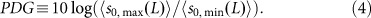


To quantify de-correlation of the pump and signal SOPs and polarisation pulling in terms of the fluctuation-induced phenomena, we introduce the RMS gain fluctuations as follows^12,14^:


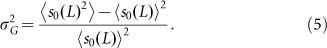


Thus, we introduced the <*G*>, PDG, and 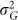 metrics to further justify the different multi-scale techniques^7,8,9,10,11,12,13,14,15,16,17,18,19,20^ using stochastic modelling.

### Vector models of the fibre Raman amplifier and multi-scale techniques

Here, we present two analytical models (where different averaging techniques have been used) and stochastic equations to validate these models. In the first model, the generic multi-scale technique has been applied, where only the randomly varying birefringence scale has been considered as the fastest scale^4,5,6^. Next, we average the fast birefringence fluctuations (details are found in the Supplementary Information) and neglect the pump depletion, cross-phase and self-phase modulations (XPM and SPM) and time dependence, i.e. group velocity dispersion (GVD). This approximation is valid for the pump powers *P*_in_ <1 W, signal powers *s*_0_ <10 mW^12,21^, *D_p_*> 0.01 ps km^−1/2^^12^. It has been estimated^12^ that the GVD can be neglected when the fibre length *L* is much smaller than the dispersion length 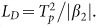 For pulse duration *T_p_* = 2.5 ps, |β_2_| = 5 ps^2^ km^−2^, we have *L_D_*> 100 km. Thus, GVD can be neglected for *L* <20 km^12^.

Taking into consideration Equation (2), we obtain the following equations, which describe the evolution of pump and signal SOPs, which are related to the pump–signal SOPs coupling:


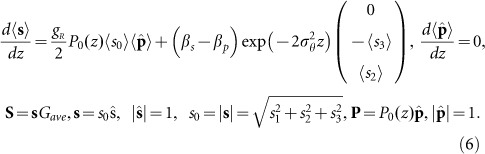


Thus, the multi-scale method includes averaging of the fast birefringence fluctuations and results in the averaged gain value and in the absence of pump and signal SOPs correlation. The method neglects gain fluctuations. Thus, one condition for the validity of the method is the low gain fluctuations. Equation (6) has been developed using unitary transformation to exclude the pump SOP fluctuations due to the random birefringence. The applied transformation preserves the length of the pump and signal SOP vectors as well as the scalar and vector products. As a result, evolution of the signal SOP includes a term (the second one), which accounts for the relative rotation of the signal SOP with respect to the pump SOP. However, Kozlov and co-workers^7^ have applied unitary transformations to the pump and signal SOPs to exclude both the pump and signal SOP fluctuations due to random birefringence and, as a result, have obtained equations that differ from Equation (6) and those derived by Sergeyev and co-workers^13^. The stimulated Raman scattering and XPM introduce a coupling between the pump and signal SOPs. Thus, the adopted transformations^7^ do not preserve either the vector and scalar lengths or the vector products.

To justify the multi-scale method that results in Equation (6), we use stochastic equations derived from the coupled Manakov-PMD equations to calculate the part of the gain, which is related to the pump–signal SOPs coupling, gain fluctuations and correlation properties of signal and pump SOPs (details are found in Supplementary Information)^12^:


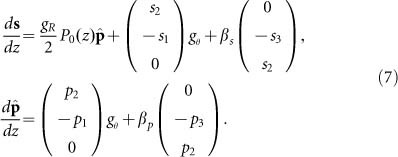


Here *g_θ_* is defined in Equation (1). Direct averaging of the randomly varying birefringence, including the scale of signal and pump waves interaction, results in the following equations^13,14,15,16,17,18,19,20^:


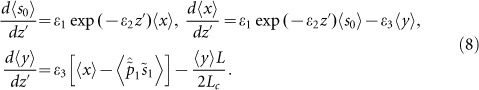


Here 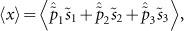 
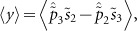
 *z*′=*z*/*L*, 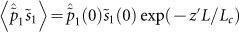, *ε*_1_=*gP*_in_*L*/2, *ε*_2_=*α_s_L*, *ε*_3_=2π*L*/*L_bp_*(*λ_s_*/λ*_p_*–1)

We also find the RMS gain fluctuations from Equation (5) using the following equations^17^:


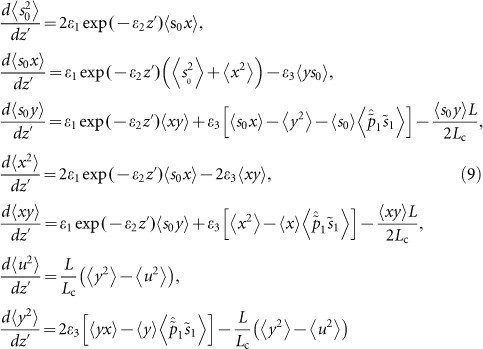


To justify validity of the multi-scale technique, we calculate gain 〈*G*〉, RMS, PDG and correlation between the pump and signal SOPs by solving Equations (6)–(9).

To quantify polarisation pulling and escape in terms of the FIE phenomena, we use the previously suggested approach^17^ to calculate parameters, which are equivalent to parameters used in the excitable systems^29^ models (the Kramers length <*L_K_*> and intrawell relaxation length <*L_R_*>). First, Equation (8) is simplified as follows:


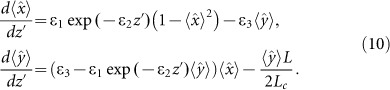


Here, 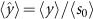 and 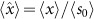 are the variables that indicate polarisation pulling if 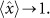 Escape from the pulling is achieved if 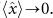 For (–*ε*_2_*z*′)≪1, the solutions of Equation (10) 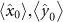 are independent of *z*′ and, thus, are derived from^17^:


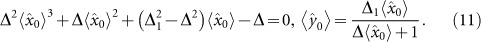


Here, Δ=2*L_c_ ε*_1_ exp(–*ε*_2_*z*′)/*L*,Δ_1_=2*L_c_ ε*_3_/*L*. Using the linear stability analysis of Equation (10) near 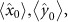 we find eigenvalues:


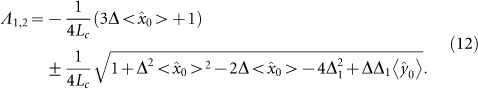


We introduce the intrawell relaxation length *L_R_*=1/|Re(*Λ*_1,2_)|. If Im(*Λ*_1,2_)≠0, the system escapes by oscillating around the states 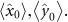 Thus, we define the Kramers length as *L_K_*=2π*L*/|Im(*Λ*_1,2_)|.

To study the long-range memory effects for the Raman-induced polarisation pulling and escape, we provide the Hurst rescale range R/S analysis to obtain the Hurst parameter^3,35,36,37,38,39,40,41,42,43,44,45,46^. First, for time series *X_i_* (*i* = 1,2…*N*) the mean value *µ_N_* and the cumulative deviate series *Γ_N,k_* are calculated as follows^3^:


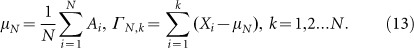


Next, the range *R_N_* and the standard deviation *S_N_* are calculated as


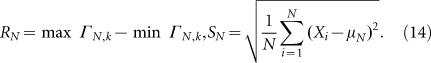


The rescale range is found as *R_N_*/*S_N_*. Then, the time series of *N* points are divided into two *N*/2-points time series, and the rescale range *R_N_*_/2_/*S_N_*_/2_ is calculated for both time series and is, then, averaged. This process is repeated for partial series which comprise *n* = *N*/4, *N*/8,… points. The Hurst parameter *H* is estimated by fitting the power law of averaged *R_n_*/*S_n_* for *n*→∞, e.g.


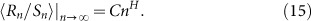


It has been determined by many authors^3,35,36,37,38,39,40,41,42,43,44,45,46^ that the Hurst parameter varies as 0 <*H* <1. The parameter 0.5 <*H* <1 is related to the persistent statistics. Thus, a positive increment in the past makes it more probable to have a positive trend in the future. This conclusion works in reverse for the anti-persistent statistics. Specifically, a positive increment in the past will result in a more probable negative trend in the future. Hurst first suggested an application of the R/S analysis to study water storage in the Nile River^36,37^. Since then, the Hurts parameter has been proven as a feasible metric for analysing long-range dependence in network traffic^39,40^, turbulence^41^, heartbeat^42^, coalition of neurons dynamics^43^, detection of low observable targets within sea clutter^44^, identification and prediction of epileptic seizures, earthquakes, and crashes in financial market^3,45,46^.

## Results and Discussion

**Figure 2:**
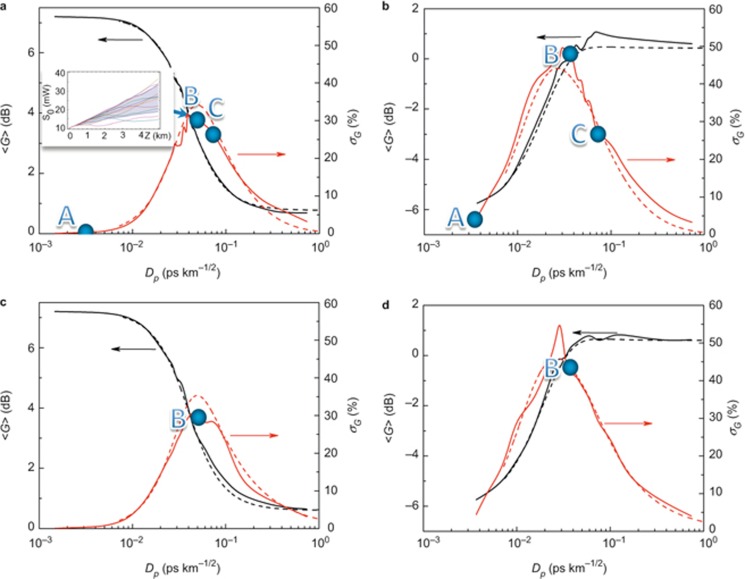
The gain <*G*> (part of the Raman gain, which is related to the pump–signal SOPs coupling), which is averaged for 100 stochastic trajectories (solid black curves), and the corresponding RMS gain fluctuations σ*_G_* (solid red curve) in comparison with the <*G*> (black dashed curves) and RMS gain fluctuations (red dashed curves) as a function of the PMD parameter *D_p_*. (**a**) and (**b**), (**c**) and (**d**) plots correspond to the Stokes parameters of the pump and the input signal fields as follows: 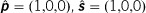 (**a**); 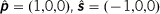 (**b**); 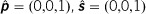 (**c**); 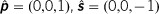 (**d**). Points *A*, *B* and *C* correspond to the signal beat lengths *L_bs_* of 200, 20 and 10 m, respectively. Inset: ten stochastic trajectories of the signal power *s_0_*. The orange dashed curve shows the average values of *s*_0_. The dashed domains demonstrate ranges of the *s*_0_ standard deviation. The parameters correspond to point *B* and 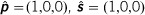. The gain <*G*> is normalised to *G*_ave_ in agreement with Equation (3).

Equation (7) have been solved using the Wolfram Mathematica 9.0 computer algebra system using the built-in Klöden–Platen–Schurz method, which provided the fastest convergence compared with the Runge–Kutta and Milstein algorithms (see the Supplementary Information). The averaging procedure was performed for an ensemble of *N* = 100 stochastic trajectories. This provides the precision of 1/*N*^1/2^ ∼ 10%, which is sufficient to justify the analytical results obtained by Sergeyev and co-workers^13,14,15,16,17,18,19,20^. We used the following parameters: the Raman gain coefficient *g* was 0.8 W^−1^ km^−1^; the input signal *s*_0_ and the pump *P_in_* powers were 10 mW and 1 W, respectively; the fibre length *L* was 5 km; the correlation length of birefringence vector *L*_c_ was 100 m. The Stokes parameters for the pump and the input signal fields, which correspond to the maximum and minimum PDG values, were: 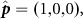 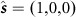 for the maximum gain and maximum PDG; 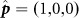, 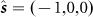 for the minimum gain and maximum PDG; 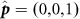, 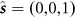 for the maximum gain and minimum PDG; and 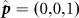, 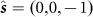 for the minimum gain and minimum PDG. Based on our previous publications and on Equations (7), the Poincaré sphere reference frame is chosen to have a local birefringence as 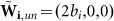 for all stochastic realisations. Thus, all trajectories for the Stokes parameter *s*_0_ have the same starting point with respect to the chosen reference frame with further divergence being caused by the random birefringence fluctuations, as shown in the Figure 2 inset.

The dependences of gain 〈*G*〉, which, according to Equation (3), is part of the Raman gain, are related to the pump–signal SOPs coupling (black solid and dashed curves), and RMS gain fluctuations σ_G_ (red solid and dashed curves) on the PMD parameter are shown in Figure 2. The solid and dashed curves correspond to the numerical solution of Equations (7)–(9). Based on Figure 2, the stochastic calculations of the gain and RMS gain fluctuations, which are derived using Equation (7), perfectly fit in the whole range of the PMD parameters using results from our previously developed model, which is based on Equations (8) and (9)^13,14,15,16,17,18,19,20^. This is a significant result because it provides analysis tools for long fibre communication systems without using time-consuming calculations, which are based on the solution of the underlying stochastic equations.

Based on Equation (6), we conclude that in view of the exponential decay of the term related to escape from polarisation pulling, an application of the multi-scale technique with averaging, excluding scale of the pump–signal SOPs interactions, results in polarisation pulling in all ranges of the PMD parameters. As a result, the gain values coincide with gain values obtained from Equations (7)–(9) in the limit of *D_p_*→0 and are close to the values for an ideal Raman polarizer^11^, by taking into account normalisation of <*G*> to *G*_ave_ in Equation (3). However, the averaging technique, which accounts for the signal and pump SOPs interaction^13,14,15,16,17,18,19,20^ scale, better agrees with the stochastic modelling results (Figure 2). In addition, these analytical techniques, which resulted in Equations (8) and (9), predict a resonant enhancement of the RMS gain fluctuations within the range of PMD parameters of 10^−2^ to 10^−1^ ps km^−1/2^, which are typical for the modern single mode fibres (the red dashed curves in Figure 2 in comparison with the red solid curves, which are obtained numerically from Equation (7)).

**Figure 3:**
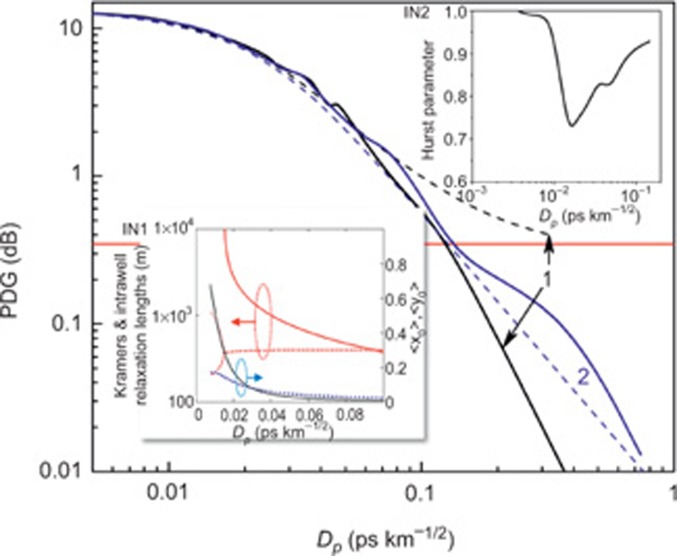
Relationship of the polarization-dependent gain (PDG) to the PMD parameter *D_p_*. Solid curves correspond to the numerical results: 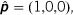 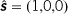 vs.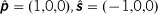 (black solid curve 1) and 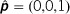, 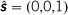 vs.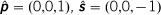 (blue solid curve 2). Analytical results^13,14^ are represented using the dashed curves (the dashed curves 1 and 2, respectively). The red solid curve represents the PDG asymptotical threshold^13,14^. Inset 1 (IN1): Results of calculation of the Kramers (red solid line) and relaxation (red dotted and dashed lines) lengths as a function of the PMD parameter *D_p_* (Equations (11) and (12)); the steady state solutions for 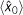 (black solid line) and 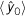 (blued dotted line). Inset 2 (IN2): dependence of the Hurst parameter for 〈*x*〉 on the PMD parameter *D_p_* 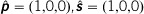.

Though the analytic theory predicts a constant asymptotic of 0.34 dB for the PDG parameter (red curve in Figure 3), the numerical PDG disappears approximately monotonically with PMD (solid curves in Figure 3), which is in agreement with the Ref. 10 results. Nevertheless, the averaging of the *N* = 100 trajectories provides a precision Δ∼1/*N*^1/2^ of 0.4 dB. Thus, the asymptotic cannot be validated for the parameters used here.

To characterise the transition from polarisation pulling to escape from pulling, we determined Kramers and the intrawell relaxation lengths from Equations (11) and (12) and the Hurst parameter for the pump-to-signal SOP projection 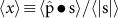 (insets 1 and 2 in Figure 3). As follows from Figure 3 (inset 1), the transition from polarisation pulling to escape has the threshold at *D_p_* ≈ 0.02 ps km^−1/2^, which, according to Equation (12), corresponds to the escape rate |Im(*Λ*_1,2_)|≥0 for <*x*> evolving along the fibre length. In contrast to our previous results on SR in the fibre Raman amplification^17^, here the escape from polarisation pulling happens in many uncorrelated steps rather than in one step, as for SR. Therefore, we have increased gain fluctuations instead of increased the signal-to-noise ratio, e.g., the stochastic anti-resonance^17^.

**Figure 4:**
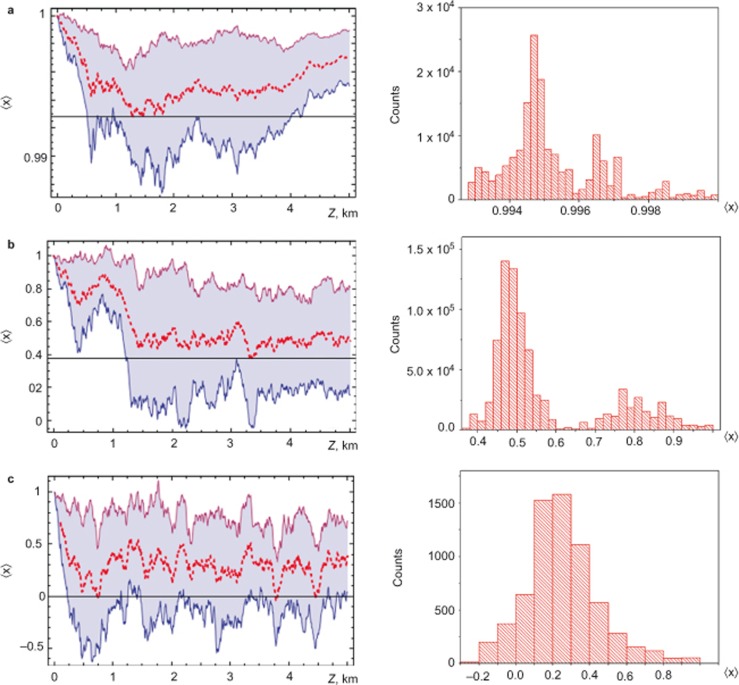
Left column: Evolution of the averaged projection 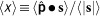 (red dashed curve) and its standard deviation (filled area) for the input gain and the signal 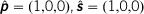 (a “maximum gain”, (**a**) in Figure 2) with the *A*-, *B*-, *C*-PMD parameters of Figure 2. Right column: the corresponding (*A*, *B* and *C*) histograms.

**Figure 5:**
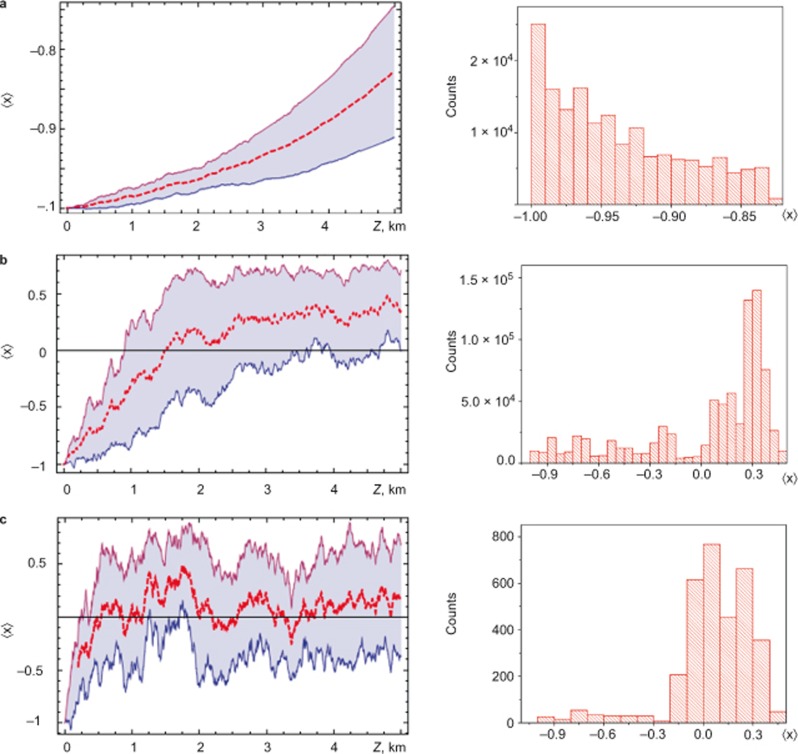
As in Figure 4, but for 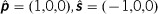
(a “minimum gain”, (**b**) in Figure 2).

**Figure 6:**
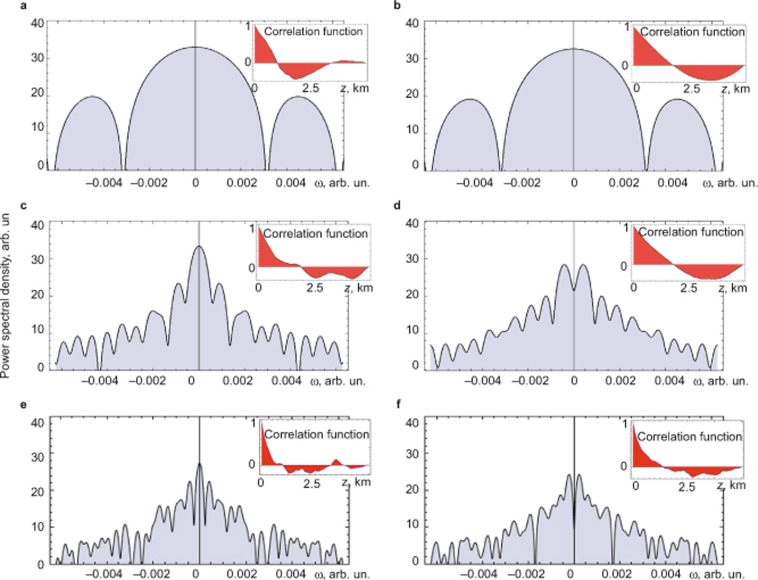
The power spectral densities of 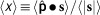
at *L* = 5 km and the *z*-dependences of the corresponding correlation function (insets) for 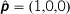, 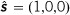 (a “maximum gain”; (**a**), (**c**) and (**e**) graphs) and 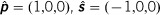 (a “minimum gain”; (**b**), (**d**) and (**f**) graphs). The PMD parameters correspond to points *A* (upper row of the graphs in Figure 6), *B* (middle row of the graphs in Figure 6) and *C* (bottom row of the graphs in Figure 6) in Figure 2.

To gain insight into statistical properties of the pump and signal SOPs interaction, we studied the stochastic evolution of the signal-to-pump SOP projection 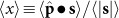 along the fibre instead of the *s*_0_ evolution (Figures 4–6). By comparing Figures 3–6 with Figure 2 (inset) and Equation (8) (first row), we determine that the evolution of <*x*> reflects the statistics of SOP interactions by including small scales, whereas for *s*_0_ the small-scale statistics disappears due to the propagation distance averaging. The asymptotic behaviour (*D_p_*→0) of <*x*> demonstrates the Raman-induced polarisation pulling effect^7,8,9,10,11,16,17,18,19,21,22,23,24,25,26,27^, where the Raman amplification plays an effective fibre polariser role because 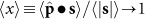, i.e., the signal SOP is attracted to the pump SOP (see the top row of Figure 4 and the black solid curve in Figure 2a). For *D_p_*→0, the fibre becomes effectively ‘isotropic’^10^. Thus, the Raman amplification anisotropy results in the strong amplification of the co-polarised to pump signal SOP and the attenuation of the cross-polarised signal SOP.

For the initially cross-polarised pump and signal SOPs, this attraction occurs (the top row of Figure 5) with a lower rate and is initiated by the birefringence fluctuations due to the escape from the metastable state with 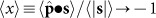. As a result, the average gain 〈*G*〉 remains minimal for the considered fibre length (Figure 2b and 2d). An important polarisation pulling property for both considered initial signal SOPs is minimisation of RMS fluctuations of the average gain (points A on the red curves in Figure 2a and 2b) and a regular structure of the spectral energy density (Figure 6a and 6b). This is due to ‘fine graining’ of the birefringence fluctuations, which play a role of white noise perturbations around a stable polarisation state (Figure 2a and 2c and the top row in Figure 4), or perturbations pulling out a metastable polarisation state (Figure 2b and 2d and the top row in Figure 5). The corresponding correlation functions demonstrate a damped oscillation behaviour (insets in Figure 6a and 6b).

The opposite extreme case is *L*_c_>>*L*_b_ (the large PMD parameters, i.e., the case of a ‘standard Raman amplifier’^10,11^), when the deterministic evolution, which is induced by the fibre birefringence, prevails over the stochastics. In this case, a single mode fibre is similar to the polarization-maintaining (PM) fibre, which has comparatively rare stochastic switches of the birefringence axis fluctuations. Thus, the RMS gain fluctuations decrease (e.g., the points C on the red curves in Figure 2a and 2b), and <*G*> approaches a constant small but non-zero value (black curves in Figure 2a and 2c). Because the evolution is driven by the fast pump–signal decorrelation, the average gain is minimal (but non-zero) for the initially co-polarised pump and signal. This means that there is a weak correlation between the pump and signal SOPs (bottom row in Figure 4).

The decrease of SOP correlation manifests itself in the Hurst parameter reduction *H* <1 (inset 2 in Figure 3). For the initially cross-polarised pump and signal SOPs, the residual correlation (bottom row in Figure 5) maximises the gain <*G*> (black curves in Figure 2b and 2d and the bottom row in Figure 5). An oscillatory evolution, which underlies localisation, along the fibre reveals itself in the modulated power spectrum densities and the corresponding correlation functions (Figure 6e and 6f).

The intermediate case of *L_b_* ≈ *L_c_*/4 (*D_p_* ≈ 10^−2^ ÷ 10^−1^ ps km^−1/2^) demonstrates a resonant enhancement of polarisation stochastic evolution, where the RMS gain fluctuations have a set of spikes (in the vicinity of points B in Figure 2). Such spikes correspond to enhanced ‘wandering’ of the trajectories for the signal-to-pump SOP projections <*x*> (Figures 4 and 5). In view of the increased rather than decreased gain fluctuations for point B, this phenomenon is contrary to the SR^28,29,30,31^ and, thus, is referred to as stochastic anti-resonance^29^. The stochasticity intensification is demonstrated by the threshold-like dropping of the Hurst parameter to *H* <0.7 and the corresponding collapse of the Kramers length (insets in Figure 3). This switching between the statistical scenarios is the distinguishing characteristic of the ‘stochastic anti-resonance’ under consideration. The average polarisation state remains ‘localised’ (the middle rows of Figures 4–5) but its sensitivity to the input SOP disappears with the PMD parameter growth (Figure 3). This means that the PDG decreases with *D_p_* (solid lines in Figure 3). Therefore, the Raman gain in the vicinity of the standard deviation peak behaves as an ‘effective depolariser’, which diminishes the PDG.

## Conclusions

In summary, using stochastic modelling, we provided insights into multi-scale polarisation phenomena for the FRA as a function of its birefringence properties (PMD parameter). We demonstrated that for the low PMD values the fibre become almost isotropic. Thus, the Raman amplification anisotropy leads to polarisation pulling when the signal SOP is attracted to the pump SOP^7,8,9,10,11,16,17,18,19,21,22,23,24,25,26,27^. Therefore, the length of the pump-to-signal SOP interaction (beat length of the relative rotation of the pump SOP with respect to the signal SOP) is much longer than the birefringence correlation length. Thus, averaging over the correlation length scale using the generic multi-scale technique^4,5,6^ leads to the results that are close to those obtained using stochastic modelling (Figures 2 and 3). Due to the decreased interaction length (increase in PMD), deterministic rotation of the signal SOP with respect to the pump SOP is intensified and results in escape phenomena, which is similar to stochastic anti-resonance, in view of the increased RMS gain fluctuations (Figure 2). When the rotation rate approaches the correlation length, the scale averaging of the correlation length is no longer valid and cannot provide correct results for the gain (Figure 2). Only by including the scale of the signal-to-pump SOPs interactions, we demonstrate that it is possible to obtain the correct results (Figures 2 and 3). A further decrease of the interaction length corresponds to an almost deterministic birefringence case, where the pump and signal SOPs rotate without interaction (Figure 2). Detailed statistical analysis of the pump-to-signal SOP projection evolution along the fibre unveiled different types of fractional Brownian motions as a function of PMD values in terms of the Hurst parameter *H*. For the low PMD values, the polarisation pulling leads to *H*→1, which corresponds to the persistent statistics. For the PMD values that correspond to the gain fluctuations maximum, the Hurst parameter decreases to *H* = 0.7 and, therefore, approaches the Brownian motion with *H* = 0.5. Further increase in the PMD parameter corresponds to the almost deterministic SOPs evolution and, thus, the persistent statistics with *H*→0.8.

The obtained results are further generalised by accounting for the pump depletion, XPM and SPM, and time dependence (GVD and walk-off between the pump and signal waves). This manipulation provides an opportunity to gain insight into the RIN^33^ and extreme statistics in the FRAs^47^ as a function of the PMD parameters and to adapt the developed methods to characterise the parametric^48^ and Brillouin^49^ amplifiers. Additionally, these results can be applied, in the context of new multi-scale methods development, to study the complex nonlinear coupled systems, such as lasers (multimode, mode-locked, random)^50,51,52^, nanostructures (light-mediated conformation of molecules and chemical reactions, Brownian motors^53^), and other systems^39,40,41,42,43,44,45,46^.

## References

[bib1] Poincaré H. Les méthodes nouvelles de la mécanique céleste, tome I. Paris: Gauthier-Villars; 1892.

[bib2] Nayfeh AH. Perturbation Methods. New York: Wiley; 1973.

[bib3] Gao J, Cao Y, Tung W, Hu J. Multiscale Analysis of Complex Time Series: Integration of Chaos and Random Fractal Theory, and Beyond. Hoboken, NJ: Wiley-Blackwell; 2007.

[bib4] Wai PKA, Menyuk CR. Polarization mode dispersion, decorrelation, and diffusion in optical fibers with randomly varying birefringence. J Lightwave Technol 1996; 14: 148–157.

[bib5] Menyuk CR. Application of multiple-length-scale methods to the study of optical fiber transmission. J Eng Math 1999; 36: 113–136.

[bib6] Menyuk CR, Marks BS. Interaction of polarization mode dispersion and nonlinearity in optical fiber transmission systems. J Lightwave Technol 2006; 24: 2806–2826.

[bib7] Kozlov VV, Nuño J, Ania-Castañón JD, Wabnitz S. Theory of fiber optic Raman polarizers. Opt Lett 2010; 35: 3970–3972. 21124582 10.1364/OL.35.003970

[bib8] Kozlov VV, Nuño J, Ania-Castañón JD, Wabnitz S. Multichannel Raman polarizer with suppressed relative intensity noise for wavelength division multiplexing transmission lines. Opt Lett 2012; 37: 2073–2075. 22660125 10.1364/OL.37.002073

[bib9] Kozlov VV, Nuño J, Ania-Castañón JD, Wabnitz S. Theoretical study of optical fiber Raman polarizers with counterpropagating beams. J Lightwave Technol 2011; 29: 341–347.

[bib10] Kozlov VV, Nuño J, Ania-Castañón JD, Wabnitz S. Trapping polarization of light in nonlinear optical fibers: an ideal Raman polarizer. In: Malomed BA, editor. Progress in Optical Science and Photonics: Spontaneous Symmetry Breaking, Self-Trapping, and Josephson Oscillations. Berlin: Springer; 2012. pp227–246.

[bib11] Kozlov VV, Nuño J, Ania-Castañón JD, Wabnitz S. Analytic theory of fiber-optic Raman polarizers. Opt Express 2012; 20: 27242–27247. 10.1364/OE.20.027242 23187579

[bib12] Lin Q, Agrawal GP. Vector theory of stimulated Raman scattering and its application to fiber-based Raman amplifiers. J Opt Soc Am B 2003; 20: 1616–1631.

[bib13] Sergeyev S, Popov S, Friberg AT. Modeling polarization-dependent gain in fiber Raman amplifiers with randomly varying birefringence. Opt Commun 2006; 262: 114–119.

[bib14] Sergeyev S, Popov S, Friberg AT. Polarization-dependent gain and gain fluctuations in a fiber Raman amplifier. J Opt A: Pure Appl Opt 2007; 9: 1119–1122.

[bib15] Sergeyev S. Fiber Raman amplifier. US Patent 2009; 12/468: 514.

[bib16] Sergeyev S, Popov S. Two-section fiber optic Raman polarizer. IEEE J Quantum Electron 2012; 48: 56–60.

[bib17] Sergeyev SV. Activated polarization pulling and de-correlation of signal and pump states of polarization in fiber Raman amplifier. Opt Express 2011; 19: 24268–24279. 22109453 10.1364/OE.19.024268

[bib18] Sergeyev S, Popov S, Friberg AT. Spun fiber Raman amplifiers with reduced polarization impairments. Opt Express 2008; 16: 14380–14389. 18794973 10.1364/oe.16.014380

[bib19] Sergeyev S. Fiber Raman amplification in a two-scale spun fiber. Opt Mater Express 2012; 2: 1683–1689.

[bib20] Sergeyev S, Popov S, Friberg AT. Virtually isotropic transmission media with fiber Raman amplifier. IEEE J Quantum Electron 2010; 46: 1492–1497.

[bib21] Ursini L, Santagiustina M, Palmieri L. Raman nonlinear polarization pulling in the pump depleted regime in randomly birefringent fibers. IEEE Photonics Technol Lett 2011; 23: 254–256.

[bib22] Chiarello F, Ursini L, Palmieri L, Santagiustina M. Polarization attraction in counterpropagating fiber Raman amplifiers. IEEE Photonics Technol Lett 2011; 23: 1457–1459

[bib23] Martinelli M, Cirigliano M, Ferrario M, Marazzi L, Martelli P. Evidence of Raman-induced polarization pulling. Opt Express 2009; 17: 947–955. 19158910 10.1364/oe.17.000947

[bib24] Popov S, Sergeyev S, Friberg AT. The impact of pump polarization on the polarization dependence of Raman gain due to the break of fiber circular symmetry. J Opt A: Pure Appl Opt 2004; 6: S72–S76.

[bib25] Muga NJ, Ferreira MFS, Pinto AN. Broadband polarization pulling using Raman amplification. Opt Express 2011; 19: 18707–18712. 21935240 10.1364/OE.19.018707

[bib26] Morin P, Pitois S, Fatome J. Simultaneous polarization attraction and Raman amplification of a light beam in optical fibers. J Opt Soc Am B 2012; 29: 2046–2052.

[bib27] Chiarello F, Palmieri L, Santagiustina M, Gamatham R, Galtarossa A. Experimental characterization of the counter-propagating Raman polarization attraction. Opt Express 2012; 20: 26050–26055. 23187420 10.1364/OE.20.026050

[bib28] Hanggi P. Escape from a metastable state. J Stat Phys 1986; 42: 105–148.

[bib29] Lindner B, Garsia-Ojalvo J, Neiman A, Schimansky-Greif L. Effects of noise in excitable systems. Phys Rep 2004; 392: 321–424.

[bib30] Gammaitoni L, Hänggi P, Jung P, Marchesoni F. Stochastic resonance. Rev Mod Phys 1998; 70: 223–287.

[bib31] Dykman MI, Golding B, McCann LI, Smelyanskiy VN, Luchinsky DG et al. Activated escape of periodically driven systems. Chaos 2001; 11: 587–594. 12779496 10.1063/1.1380368

[bib32] Foschini GJ, Poole CD. Statistical theory of polarization dispersion in single mode fibers. J Lightwave Technol 1991; 9: 1439–1456.

[bib33] Chang D, Pelouch W, Perrier P, Fevrier H, Ten S et al.150 × 120 Gb/s unrepeatered transmission over 409.6 km of large effective area fiber with commercial Raman DWDM system. Opt Express 2014; 22: 31057–31062. 25607055 10.1364/OE.22.031057

[bib34] Martinelli C, Lorcy L, Durecu-Legrand A, Mongardien D, Borne S et al. RIN transfer in copumped Raman amplifiers using polarization-combined diodes. IEEE Photonics Technol Lett 2005; 17: 1836–1838.

[bib35] Meyers RA (ed). Mathematics of Complexity and Dynamical Systems. New York: Springer; 2009.

[bib36] Hurst H. Long term storage capacity of reservoirs. Trans Am Soc Civ Eng 1951; 116: 770–808.

[bib37] Hurst HE, Black RP, Simaika YM. Long-term Storage: An Experimental Study. London: Constable; 1965.

[bib38] Feder J. Fractals. New York: Plenum Press; 1988, pp149–183.

[bib39] Abry P, Veitch D. Wavelet analysis of long-range-dependent traffic. IEEE Trans Inf Theory 1998; 44: 2–15.

[bib40] Resta M. Hurst exponent and its applications in time-series analysis. Recent Pat Comput Sci 2012; 5: 211–219.

[bib41] Frisch H. Turbulence – The Legacy of A. N. Kolmogorov. Cambridge: Cambridge University Press; 1995.

[bib42] Ivanov PC, Amaral LAN, Goldberg AL, Havlin Sh, Rosenblum MG et al. Multifractality in human heartbeat dynamics. Nature 1999; 399: 461–465. 10365957 10.1038/20924

[bib43] Zhou YH, Gao JB, White KD, Merk I, Yao K. Perceptual dominance time distributions in multistable visual perception. Bio Cybern 2004; 90: 256–263. 15085344 10.1007/s00422-004-0472-8

[bib44] Hu J, Tung WW, Gao JB. Detection of low observable targets sea clutter by structure function based multifractal analysis. IEEE Trans Antennas Propag 2006; 54: 136–143.

[bib45] Eftaxias K, Minadakis G, Potirakis SM, Balasis G. Dynamical analogy between epileptic seizures and seismogenic electromagnetic emissions by means of nonextensive statistical mechanics. Physica A 2013; 392: 497–509.

[bib46] Grech D, Mazur Z. Can one make any crash prediction in finance using the local Hurst exponent idea? Physica A 2004; 336: 133–145.

[bib47] Hammani K, Picozzi A, Finot Ch. Extreme statistics in Raman fiber amplifiers: from analytical description to experiments. Opt Commun 2011; 284: 2594–2603.

[bib48] Guasoni M, Kozlov VV, Wabnitz S. Theory of polarization attraction in parametricamplifiers based on telecommunication fibers. J Opt Soc Am B 2012; 29: 2710–2720.

[bib49] Galtarossa A, Palmieri L, Santagiustina M, Schenato L, Ursini L. Polarized Brillouin amplification in randomly birefringent and unidirectionally spun fibers. IEEE Photonics Technol Lett 2008; 20: 1420–1422.

[bib50] Turitsyn SK, Babin SA, El-Taher AE, Harper P, Churkin DV et al. Random distributed feedback fibre laser. Nat Photonics 2010; 4: 231–235.

[bib51] Turitsyna EG, Smirnov SV, Sugavanam S, Tarasov N, Shu X et al. The laminar-turbulent transition in a fibre laser. Nat Photonics 2013; 7: 783–786.

[bib52] Sergeyev SV, Mou C, Turitsyna EG, Rozhin A, Turitsyn SK et al. Spiral attractor created by Vector Solitons. Light: Sci Appl 2014; 3: e131; doi:10.1038/lsa.2014.12.

[bib53] Hänggi P, Marchesoni F. Artificial Brownian motors: controlling transport on the nanoscale. Rev Mod Phys 2009; 81: 387–442.

