# Supplementary material for: Multi-scale polarisation phenomena
Source: Light Sci Appl. 2016 Jan 15;5(1):e16011–. doi: 10.1038/lsa.2016.11 (PMC6059843; doi:10.1038/lsa.2016.11)
Supplement: Supplementary Information [file lsa201611x1.docx]

**Multi-scale polarisation phenomena**

Vladimir Kalashnikov^1^, Sergey V. Sergeyev^1*^, Gunnar Jacobsen^2^, Sergei Popov^3^ and Sergei K. Turitsyn^1^

^1^ AIPT, Aston University, B4 7ET, Birmingham, UK

^2^ Acreo, Electrum 236, SE-16440, Kista, Sweden

^3^ Royal Institute of Technology, SE-1640, Stockholm, Sweden

^*^Contact: s.sergeyev@aston.ac.uk

**SUPPLEMENTARY INFORMATION**

In the paper “Multi-scale polarisation phenomena”, we have modelled a stochastic alteration of statistical properties of polarisation phenomena in a fibre Raman amplifier and demonstrated a transit from the polarisation pulling to the resonance-like escape from the pulling. In this Supplementary paper we present an application of a widely used multi-scale technique for derivation of coupled Manakov’s equations describing evolution of the state of polarisations for pump and signal waves in a fibre Raman amplifier.

**Multi-scaling technique**

We start from the coupled Manakov-PMD equations describing co-propagation signal and pump waves in a fibre Raman amplifier [1-5]:

(1)

Where

$\sigma_{1}=\left[\begin{matrix} 0 & 1 \\ 1 & 0 \end{matrix}\right], \sigma_{2}=\left[\begin{matrix} 0 & -i \\ i & 0 \end{matrix}\right], \sigma_{3}=\left[\begin{matrix} 1 & 0 \\ 0 & -1 \end{matrix}\right], |A_{i}\rangle=\left(A_{ix},A_{iy}\right)^{T}, \langle A_{i}|=|A_{i}\rangle^{+}=\left(A_{ix}^{*},A_{iy}^{*}\right)$ (2)

By using rotation matrix *R* as follows [3-5]:

(3)

We find:

$\begin{matrix} i\frac{\partial|a_{s}\rangle}{\partial z}+\Sigma_{s}|a_{s}\rangle+i\frac{\alpha_{s}}{2}|a_{s}\rangle+i\Delta\beta_{s}^{'}\sigma_{3}\frac{\partial|a_{s}\rangle}{\partial t}-\frac{1}{2}\Delta\beta_{s}^{''}\frac{\partial^{2}|a_{s}\rangle}{\partial t^{2}}+\frac{\gamma_{ss}}{3}\left(2\langle a_{s}||a_{s}\rangle+|a_{s}^{*}\rangle\langle a_{s}^{*}|\right)|A_{s}\rangle+ \\ \frac{2\gamma_{sp}}{3}\left(2\langle a_{p}||a_{p}\rangle+|a_{p}\rangle\langle a_{p}|+|a_{p}^{*}\rangle\langle a_{p}^{*}|\right)|a_{s}\rangle-\frac{ig_{R}}{2}|a_{p}\rangle\langle a_{p}||a{}_{s}\rangle=0, \\ i\frac{\partial|a_{p}\rangle}{\partial z}+\Sigma_{p}|a_{p}\rangle+i\frac{\alpha_{p}}{2}|a_{p}\rangle+i\Delta\beta_{p}^{'}\sigma_{3}\frac{\partial|a_{p}\rangle}{\partial t}-\frac{1}{2}\Delta\beta_{p}^{''}\frac{\partial^{2}|a_{p}\rangle}{\partial t^{2}}+ \\ +\frac{\gamma_{pp}}{3}\left(2\langle a_{p}||a_{p}\rangle+|a_{p}^{*}\rangle\langle a_{p}^{*}|\right)|a_{p}\rangle+\frac{2\gamma_{sp}}{3}\left(2\langle a_{s}||a_{s}\rangle+|a_{s}\rangle\langle a_{s}|+|a_{s}^{*}\rangle\langle a_{s}^{*}|\right)|a_{p}\rangle- \\ -\frac{ig_{R}}{2}\frac{\omega_{p}}{\omega_{s}}|a_{s}\rangle\langle a_{s}||a{}_{p}\rangle=0. \end{matrix}$(4)

Where

$\Sigma_{i}=\left[\begin{matrix} \Delta\beta_{i} & -\theta_{z} \\ \theta_{z} & -\Delta\beta_{i} \end{matrix}\right], \theta_{z}\equiv\frac{\partial\theta}{\partial z}=g_{\theta}\left(z\right), \left\langle g_{\theta}\left(z\right)\right\rangle=0, \left\langle g_{\theta}\left(z\right)g_{\theta}\left(z^{'}\right)\right\rangle=\sigma_{\theta}^{2}\delta\left(z-z^{'}\right)$. (5)

By using unitary transformation *T* [3-5]

(6)

We find

(7)

Here

(8)

Here

(9)

And equations for *Ω_i_* take the form:

$\begin{matrix} \frac{d\mathbf{\Omega}}{dz}=\mathbf{Q}g_{\theta}(z)+\mathbf{\Lambda}, \\ \mathbf{Q}=\left(Q_{1},Q_{2},Q_{3},Q_{4},Q_{5},Q_{6}\right)^{T}, \mathbf{\Lambda}=\left(\Lambda_{1},\Lambda_{2},\Lambda_{3},\Lambda_{4},\Lambda_{5},\Lambda_{6}\right)^{T} \\ \left\langle g_{\theta}\left(z\right)\right\rangle=0, \left\langle g_{\theta}\left(z\right)g_{\theta}\left(z^{'}\right)\right\rangle=\sigma_{\theta}^{2}\delta\left(z-z^{'}\right), \\ \Lambda_{1}=0, \Lambda_{2}=-2\Delta\beta_{p}\Omega_{3}, \Lambda_{3}=2\Delta\beta_{p}\Omega_{2}, \Lambda_{4}=0, \Lambda_{5}=-2\Delta\beta_{p}\Omega_{6}, \Lambda_{6}=2\Delta\beta_{p}\Omega_{5}, \\ Q_{1}=2\Omega_{2}, Q_{2}=-2\Omega_{1}, Q_{3}=0, Q_{4}=2\Omega_{5}, Q_{5}=-2\Omega_{4}, Q_{6}=0. \end{matrix}$(10)

For averaging over random birefringence we use the following notations [3-5]

$\frac{d\left\langle\psi\left(\Omega_{i}\right)\right\rangle}{dz}=\left\langle\mathrm{G}\left[\psi\left(\Omega_{i}\right)\right]\right\rangle,\mathrm{G}\left[...\right]=\sum_{i=1}^{6} \Lambda_{i}\frac{\partial}{\partial\Omega_{i}}+\frac{\sigma_{\theta}^{2}}{2}\sum_{i=1}^{6} \sum_{k=1}^{6} \left(Q_{i}Q_{k}+Q_{k}\frac{\partial Q_{i}}{\partial\Omega_{k}}\frac{\partial}{\partial\Omega_{i}}\right)$. (11)

In further notations we skip brackets for averaging <…> and so obtain:

$\begin{matrix} \frac{d\Omega_{1}}{dz}=-2\sigma_{\theta}^{2}\Omega_{1}, \frac{d\Omega_{2}}{dz}=-2\Delta\beta_{p}\Omega_{3}-2\sigma_{\theta}^{2}\Omega_{2}, \frac{d\Omega_{3}}{dz}=2\Delta\beta_{p}\Omega_{2}, \\ \frac{d\Omega_{4}}{dz}=-2\sigma_{\theta}^{2}\Omega_{4}, \frac{d\Omega_{5}}{dz}=-2\Delta\beta_{p}\Omega_{6}-2\sigma_{\theta}^{2}\Omega_{5}, \frac{d\Omega_{6}}{dz}=2\Delta\beta_{p}\Omega_{5}, \\ \left(\Omega_{1}, \Omega_{2}, \Omega_{3}, \Omega_{4}, \Omega_{5}, \Omega_{6}, \right)\left|_{0}\right.=\left(1,0,0,0,0,0\right). \end{matrix}$ (12)

As a result: $\Omega_{1}\left(z\right)=\exp\left(-2\sigma^{2}z\right) .$

$\begin{matrix} \frac{d\Omega_{1}^{2}}{dz}=4\sigma_{\theta}^{2}\left(\Omega_{2}^{2}-\Omega_{1}^{2}\right), \\ \frac{d\Omega_{2}^{2}}{dz}=-4\Delta\beta_{p}\Omega_{2}\Omega_{3}+4\sigma_{\theta}^{2}\left(\Omega_{1}^{2}-\Omega_{2}^{2}\right), \\ \frac{d\Omega_{3}^{2}}{dz}=4\Delta\beta_{p}\Omega_{2}\Omega_{3}, \\ \frac{d\Omega_{2}\Omega_{3}}{dz}=2\Delta\beta_{p}\left(\Omega_{2}^{2}-\Omega_{3}^{2}\right)-2\sigma_{\theta}^{2}\Omega_{2}\Omega_{3}, \\ \left(\Omega_{1}^{2},\Omega_{2}^{2},\Omega_{3}^{2},\Omega_{2}\Omega_{3}\right)\left|_{0}\right.=\left(1,0,0,0\right). \end{matrix}$ (13)

$\begin{matrix} \frac{d\Omega_{4}^{2}}{dz}=4\sigma_{\theta}^{2}\left(\Omega_{5}^{2}-\Omega_{4}^{2}\right), \\ \frac{d\Omega_{5}^{2}}{dz}=-4\Delta\beta_{p}\Omega_{5}\Omega_{6}+4\sigma_{\theta}^{2}\left(\Omega_{4}^{2}-\Omega_{5}^{2}\right), \\ \frac{d\Omega_{6}^{2}}{dz}=4\Delta\beta_{p}\Omega_{5}\Omega_{6}, \\ \frac{d\Omega_{5}\Omega_{6}}{dz}=2\Delta\beta_{p}\left(\Omega_{5}^{2}-\Omega_{6}^{2}\right)-2\sigma_{\theta}^{2}\Omega_{5}\Omega_{6}, \\ \left(\Omega_{4}^{2},\Omega_{5}^{2},\Omega_{6}^{2},\Omega_{5}\Omega_{6}\right)\left|_{0}\right.=\left(0,1,-1,-i\right). \end{matrix}$ (14)

$\begin{matrix} \frac{d\left|\Omega_{4}\right|^{2}}{dz}=4\sigma_{\theta}^{2}\left(\left|\Omega_{5}\right|^{2}-\left|\Omega_{4}\right|^{2}\right), \\ \frac{d\left|\Omega_{5}\right|^{2}}{dz}=-2\Delta\beta_{p}\left(\Omega_{5}\Omega_{6}^{*}+\Omega_{6}\Omega_{5}^{*}\right)-4\sigma_{\theta}^{2}\left(\left|\Omega_{5}\right|^{2}-\left|\Omega_{4}\right|^{2}\right), \\ \frac{d\left|\Omega_{6}\right|^{2}}{dz}=2\Delta\beta_{p}\left(\Omega_{5}\Omega_{6}^{*}+\Omega_{6}\Omega_{5}^{*}\right), \\ \frac{d\left(\Omega_{5}\Omega_{6}^{*}+\Omega_{6}\Omega_{5}^{*}\right)}{dz}=4\Delta\beta_{p}\left(\left|\Omega_{5}\right|^{2}-\left|\Omega_{6}\right|^{2}\right)-2\sigma_{\theta}^{2}\left(\Omega_{5}\Omega_{6}^{*}+\Omega_{6}\Omega_{5}^{*}\right), \\ \left(\left|\Omega_{4}\right|^{2},\left|\Omega_{5}\right|^{2},\left|\Omega_{6}\right|^{2},\left(\Omega_{5}\Omega_{6}^{*}+\Omega_{6}\Omega_{5}^{*}\right)\right)\left|_{0}\right.=\left(0, 1, 1, 0\right). \end{matrix}$ (15)

$\begin{matrix} \frac{d\Omega_{3}\Omega_{6}}{dz}=2\Delta\beta_{p}\left(\Omega_{2}\Omega_{6}+\Omega_{3}\Omega_{5}\right), \\ \frac{d\left(\Omega_{2}\Omega_{6}+\Omega_{3}\Omega_{5}\right)}{dz}=4\Delta\beta_{p}\left(\Omega_{2}\Omega_{5}-\Omega_{3}\Omega_{6}\right)-2\sigma_{\theta}^{2}\left(\Omega_{2}\Omega_{6}+\Omega_{3}\Omega_{5}\right), \\ \frac{d\Omega_{2}\Omega_{5}}{dz}=-2\Delta\beta_{p}\left(\Omega_{2}\Omega_{6}+\Omega_{3}\Omega_{5}\right)-4\sigma_{\theta}^{2}\left(\Omega_{2}\Omega_{5}-\Omega_{1}\Omega_{4}\right), \\ \frac{d\Omega_{1}\Omega_{4}}{dz}=4\sigma_{\theta}^{2}\left(\Omega_{2}\Omega_{5}-\Omega_{1}\Omega_{4}\right), \\ \left(\Omega_{3}\Omega_{6},\left(\Omega_{2}\Omega_{6}+\Omega_{3}\Omega_{5}\right),\Omega_{2}\Omega_{5},\Omega_{1}\Omega_{4}\right)\left|_{0}\right.=\left(0, 0, 0, 0\right). \end{matrix}$ (16)

$\begin{matrix} \frac{d\Omega_{3}\Omega_{6}^{*}}{dz}=2\Delta\beta_{p}\left(\Omega_{2}\Omega_{6}^{*}+\Omega_{3}\Omega_{5}^{*}\right), \\ \frac{d\Omega_{2}\Omega_{6}^{*}}{dz}=2\Delta\beta_{p}\left(\Omega_{2}\Omega_{5}^{*}-\Omega_{3}\Omega_{6}^{*}\right)-2\sigma^{2}\Omega_{2}\Omega_{6}^{*}, \\ \frac{d\Omega_{2}\Omega_{5}^{*}}{dz}=-2\Delta\beta_{p}\left(\Omega_{2}\Omega_{6}^{*}+\Omega_{3}\Omega_{5}^{*}\right)-4\sigma^{2}\left(\Omega_{2}\Omega_{5}^{*}-\Omega_{1}\Omega_{4}^{*}\right), \\ \frac{d\Omega_{1}\Omega_{4}^{*}}{dz}=4\sigma^{2}\left(\Omega_{2}\Omega_{5}^{*}-\Omega_{1}\Omega_{4}^{*}\right), \\ \frac{d\Omega_{3}\Omega_{5}^{*}}{dz}=2\Delta\beta_{p}\left(\Omega_{2}\Omega_{5}^{*}-\Omega_{3}\Omega_{6}^{*}\right)-2\sigma^{2}\Omega_{3}\Omega_{5}^{*} \\ \left(\Omega_{3}\Omega_{6}^{*},\Omega_{2}\Omega_{6}^{*},\Omega_{2}\Omega_{5}^{*},\Omega_{1}\Omega_{4}^{*},\Omega_{3}\Omega_{5}^{*}\right)\left|_{0}\right.=\left(0, 0, 0, 0,0\right). \end{matrix}$ (17)

As result we find:

$$\Omega_{1}\left(z\right)=\exp\left(-2\sigma^{2}z\right), b_{1}=0, b_{3}=0, b_{2}\neq0, b_{4}\neq0, b_{5}\neq0.$$

To check a validity of averaging procedure, we consider the Raman amplification in a short piece of fibre (5 km) with low input signal power (10 mW). These conditions allow us to neglect pump depletion, cross-phase and self-phase modulation and to skip time dependence. It has been found that margins of such approximation are: pump powers *P_in_* <1 W, signal powers *P_s_* <10 mW and $D_{p}$ > 0.01 ps km^-1/2^ , pulse width > 2.5 ps for fibre length *L <20 km* [1, 6].

With accounting for these notations and by using Jones to Stokes space transformation

we arrive to

(18)

Here .

**Numerical technique**

The equations describing distributed Raman amplification with a gain coefficient *g* and a distance-dependent pump and signal powers *P_0_*(*z*), *s_0_*(*z*), respectively, as well as the unit vector components of pump and signal SOPs (*p_1_*,*p_2_*,*p_3_*) and (*s_1_*,*s_2_*,*s_3_*), respectively, can be expressed in the form of system of stochastic differential equations of Stratonovich type (Equation 7 in main text [1]):

$$\begin{matrix} P_{0}\left(z\right)=P_{in}\exp\left(-\alpha_{p}\left(L\left(1-\eta\right)/2+\eta z\right)\right), \\ ds_{0}\left(z\right)=\frac{1}{2}gP_{0}\left(z\right)x\left(z\right)s_{0}\left(z\right)dz, \\ d\left[s_{0}\left(z\right)s_{1}\left(z\right)\right]=\frac{1}{2}gP_{0}\left(z\right)s_{0}\left(z\right)p_{1}\left(z\right)dz+s_{2}\left(z\right)s_{0}\left(z\right)\circ dW\left(z\right), \\ d\left[s_{0}\left(z\right)s_{2}\left(z\right)\right]=\left[\frac{1}{2}gP_{0}\left(z\right)s_{0}\left(z\right)p_{2}\left(z\right)-2b_{s}s_{3}\left(z\right)s_{0}\left(z\right)\right]dz-s_{1}\left(z\right)s_{0}\left(z\right)\circ dW\left(z\right), \\ d\left[s_{0}\left(z\right)s_{3}\left(z\right)\right]=\left[\frac{1}{2}gP_{0}\left(z\right)s_{0}\left(z\right)p_{3}\left(z\right)+2b_{s}s_{2}\left(z\right)s_{0}\left(z\right)\right]dz, (19) \\ \eta dp_{1}\left(z\right)=p_{2}\left(z\right)\circ dW\left(z\right), \eta dp_{2}\left(z\right)=-2b_{p}p_{3}\left(z\right)dz-p_{1}\left(z\right)\circ dW\left(z\right), \\ \eta dp_{3}\left(z\right)=2b_{p}p_{2}\left(z\right)dz, \\ d\left[s_{0}\left(z\right)x\left(z\right)\right]=\left[\frac{1}{2}gP_{0}\left(z\right)s_{0}\left(z\right)-2s_{0}\left(z\right)\left(b_{p}\eta-b_{s}\right)\left(p_{3}\left(z\right)s_{2}\left(z\right)-p_{2}\left(z\right)s_{3}\left(z\right)\right)\right]dz+ \\ +\left(1-\eta\right)s_{0}\left(z\right)\left(p_{1}\left(z\right)s_{2}\left(z\right)-p_{2}\left(z\right)s_{1}\left(z\right)\right)\circ dW\left(z\right). \end{matrix}$$

Here η =+1 or -1for co- or backward pump-signal propagation, $x\left(z\right)=\hat{p}\bullet\hat{s}$ is a pump-to-signal SOP projection, and *W*(*z*) represents a Winer process with a drift 0 and volatility 1/*L_c_*.

Different algorithms for solution of Equation 19 have been tested in framework of both Wolfram Mathematica 9.0 and Matlab environments. Figure S1 demonstrates the sets of 10 stochastic trajectories with the corresponding average (dashed curve) values and standard deviation ranges (filled) for a signal power evolution obtained on the basis of different numerical techniques (Wolfram Mathematica 9.0).


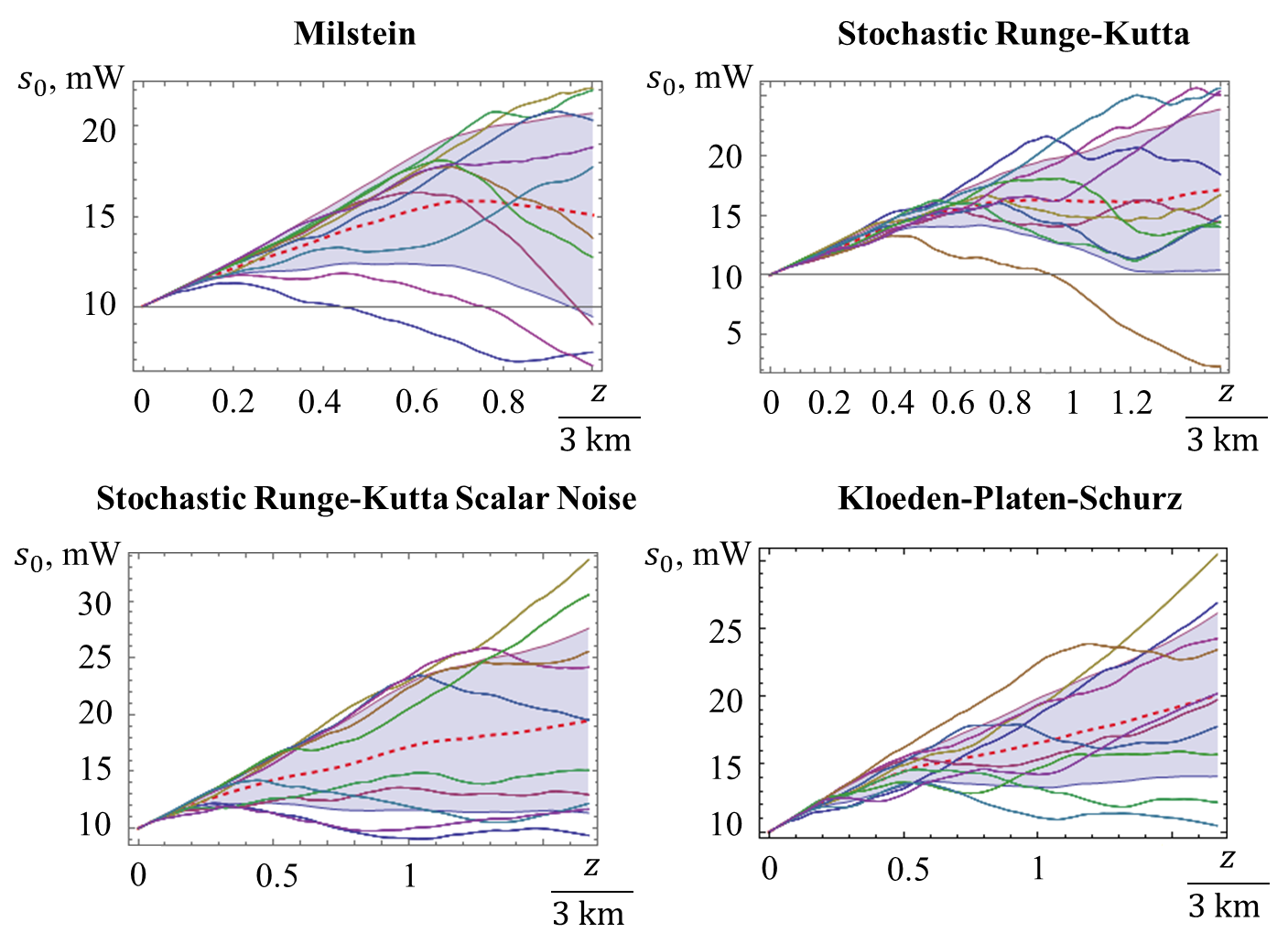


**Figure S1**. Ten stochastic trajectories of the signal power *s_0_*. The orange dashed curve shows the averaged values of *s*_0_. Filled domains demonstrate the ranges of the *s*_0_ standard deviation. The parameters correspond to the point *B* and $\hat{p}$*=*(1,0,0)*,* $\hat{s}$*=*(1,0,0) in Figure 2 in the main text*.*

Our tests have demonstrated that only algorithms shown in bottom row in Figure S1 do not produce destructively wandering stochastic trajectories which would lead to numerical divergences as it takes a place for the algorithms shown in top row (Figure S1, the algorithms shown in top row diverge). Additionally, the tests have demonstrated that the Kloeden-Platen-Schurz algorithm provides a fastest convergence in our case.

The variation of step size $\Delta z=10^{-n}\min\left(L, L_{c}, L_{bs}\right)$ within a range of *n*=3÷5 suggested that *n*=4 provides with a perfect numerical convergence for a whole diapason of PMDs considered. One has to note that a relatively large step-size (e.g., *n*<3) shifts the <*G*> and σ*_G_* curves into region of larger *D_p_*. Also, we tested a sensitivity of results obtained to a number of stochastic trajectories. We may conclude that 100 stochastic trajectories provides with a sufficient statistical precision in our case (see Figure S2).

**Figure S2.** RMS gain fluctuations $\sigma_{G}$ obtained for a different number of stochastic trajectories corresponding to the point *B* and $\hat{p}$*=*(1,0,0)*,* $\hat{s}$*=*(1,0,0) in Figure 2 in the main text*.*

**References**

1. Lin Q, Agrawal GP. Vector theory of stimulated Raman scattering and its application to fiber-based Raman amplifiers. *J Opt Soc Am B* 2003; **20**: 1616-1631.
2. Kozlov VV, Nuño J, Ania-Castañón JD, and Wabnitz S. Trapping polarization of light in nonlinear optical fibers: An ideal Raman polarizer Trapping polarization of light in nonlinear optical fibers: An ideal Raman polarizer. *In: Progress in Optical Science and Photonics: Spontaneous Symmetry Breaking, Self-Trapping, and Josephson Oscillations,* Malomed, BA (Ed.) Berlin: Springer, 2012: 227–246.
3. Wai PKA, Menyuk CR. Polarization mode dispersion, decorrelation, and diffusion in optical fibers with randomly varying birefringence. *J Lightwave Technol* 1996; **14**: 148-157.
4. Menyuk CR. Application of multiple-length-scale methods to the study of optical fiber transmission*. J Engineering Math* 1999; **36**: 113-136.
5. Menyuk CR, Marks BS. Interaction of Polarization Mode Dispersion and Nonlinearity in Optical Fiber Transmission Systems. *J Lightwave* *Technol* 2006; **24**: 2806- 2826.
6. Martinelli M, Cirigliano M, Ferrario M, Marazzi L, Martelli P. Evidence of Raman-induced polarization pulling. *Opt Express* 2009; **17**: 947-955.
